# Derotational Osteotomy of the Distal Femur for the Treatment of Patellofemoral Instability Simultaneously Leads to the Correction of Frontal Alignment: A Laboratory Cadaveric Study

**DOI:** 10.1177/2325967118775664

**Published:** 2018-06-01

**Authors:** Florian B. Imhoff, Knut Beitzel, Philip Zakko, Elifho Obopilwe, Andreas Voss, Bastian Scheiderer, Daichi Morikawa, Augustus D. Mazzocca, Robert A. Arciero, Andreas B. Imhoff

**Affiliations:** † Department of Orthopaedic Surgery, University of Connecticut Health Center, Farmington, Connecticut, USA.; ‡ Department of Orthopaedic Sports Medicine, Technical University of Munich, Munich, Germany.; § Department of Orthopaedic Surgery, Juntendo University, Tokyo, Japan.; *Investigation performed at the Department of Orthopaedic Surgery, University of Connecticut Health Center, Farmington, Connecticut, USA*

**Keywords:** distal femoral derotational osteotomy, femoral antetorsion, patellofemoral instability, valgus-varus alignment, torsion correction, 3D printing

## Abstract

**Background:**

Derotational osteotomy of the distal femur allows the anatomic treatment of patellofemoral maltracking due to increased femoral antetorsion. However, such rotational osteotomy procedures have a high potential of intended/unintended changes of frontal alignment.

**Purpose/Hypothesis:**

The purpose of this study was to perform derotational osteotomy of the distal femur and to demonstrate the utility of a novel trigonometric approach to address 3-dimensional (3D) changes on 2-dimensional imaging (axial computed tomography [CT] and frontal-plane radiography). The hypothesis was that 1-step single-cut osteotomy can simultaneously correct torsion and frontal alignment based on preoperatively calculated cutting angles.

**Study Design:**

Controlled laboratory study.

**Methods:**

Eight human cadaveric whole legs (4 lower limb torsos) underwent derotational osteotomy of the distal femur of 20°. A straight leg axis, determined as a mechanical femorotibial angle (mFTA) of 0°, was chosen as a goal for postoperative frontal alignment. The inclination of the cutting angle from the lateral view was calculated individually for each cadaveric leg and was represented by a simple 3D-printed cutting guide for surgery. Specimens underwent CT for the measurement of torsion, while the frontal leg axis was determined on an upright radiograph preoperatively and postoperatively. Preoperative and postoperative angles were compared with the mathematical prediction model.

**Results:**

The preoperative mFTA ranged from –3.9° (valgus) to +3.4° (varus) (mean, –0.2° ± 2.6°). A postoperative mean mFTA of 0.37° ± 0.69° (95% CI, –0.22° to 0.95°) was achieved (*P* = .01). Derotation showed a mean of 19.1° ± 2.1° (95% CI, 17.3°-20.8°). The oblique cutting plane for the correction of valgus legs showed a mean of 5.9° ± 6.8° and, for the correction of varus legs, a mean of –10.0° ± 4.5° projected on the perpendicular plane to the virtual anatomic shaft axis from the sagittal view.

**Conclusion:**

Single-cut distal femoral osteotomy can be performed to simultaneously address rotational as well as frontal alignment using a preoperatively defined oblique cut, as determined by the presented reproducible calculation model.

**Clinical Relevance:**

This study adds important knowledge to the technique of derotational osteotomy. This approach provides an individual, oblique single cut for the correction of torsion and frontal axis within a clinically insignificant margin. Simplified tables for calculation and a surgical reference make this model reproducible and safe.

The surgical treatment of recurrent patellofemoral instability aims to correct maltracking of the knee extensor mechanism. Besides dysplasia of the trochlea, increased tibial tubercle–trochlear groove (TT-TG) distance, and patella alta, other osseous malformations such as torsional deformity or severe frontal malalignment can be found in these cases. Increased femoral or tibial torsion is also a known risk factor in patellofemoral dislocations.^6,8,9,25^ In 1964, increased femoral antetorsion was described by Brattström^1^ as a cause of alignment maltracking in cases of patellofemoral instability. In his figures, he described supracondylar derotational osteotomy, developed by Fuermeier in 1953 and Kiesselbach in 1956, as a potential surgical treatment.^1^

Several descriptions of the surgical technique of distal femoral, or supracondylar, derotational osteotomy can be found in the recent literature.^12,19,27^ These techniques typically involve a straight single cut at the distal femur, sometimes adding a biplanar cut to improve primary fixation stability and osseous consolidation. However, none of the older or recently published techniques provide an anatomic reference for the desired cut. Rather than performing a straight single cut at the distal femur, could a defined cutting angle unique to each patient’s anatomy provide a solution to malalignment of axes that is often seen after derotational osteotomy? Paley and Herzenberg^22^ showed that the correction of frontal axis and torsion can be calculated and performed with single-cut osteotomy. This was also shown by Kim^15^ with proximal femoral osteotomy. Our study aimed to investigate a new mathematical approach for reproducible derotational osteotomy of the distal femur, as performed in cases of patellofemoral instability caused by increased femoral antetorsion, and provide a guide for anatomic reference of the cutting plane.

The purpose of this study was to perform derotational osteotomy of the distal femur and to demonstrate the utility of a novel mathematical approach to predict and address 3-dimensional (3D) changes. The hypothesis was that reproducible 1-step single-cut osteotomy with a preoperatively defined oblique angle of the cutting plane can precisely address frontal alignment after derotational osteotomy of the distal femur.

## Methods

### Specimens

Eight fresh-frozen human cadaveric whole legs were used in this study. Four lower limb torsos with a mean age of 75 years (range, 67-80 years) were obtained from Science Care. Exclusion criteria were severe knee and hip osteoarthritis and obvious severe frontal malalignment (varus or valgus angle >15°); no specimens had to be excluded. The study was reported to the institutional review board of the University of Connecticut, and it was documented that no institutional review board approval was required (deidentified specimens do not constitute human participant research). For imaging and surgery, specimens were thawed at room temperature 72 hours before testing. A 5-mm Steinmann pin was drilled through the posterior femoral condyles with a C-arm x-ray control and mounted onto a custom-made U-shaped wooden box, which allowed reproducible frontal-plane alignment of the knee on the x-ray table preoperatively and postoperatively.

### Experimental Setup

As a proof of concept, a standardized protocol was developed to investigate mathematical predictions in a laboratory cadaveric model. For consistency in this controlled laboratory setup, all specimens underwent an intended derotation (external rotation of the distal femur) of 20°. This amount was chosen because it reflects an average derotational angle that is commonly used by the authors and described in the literature.^19,27,31^ In addition, frontal alignment correction was set to achieve the goal of a straight leg axis, determined as a mechanical femorotibial angle (mFTA) of 0° for all specimens. This mathematical model is effective regardless of preoperatively detected valgus or varus alignment. Based on a new trigonometric algorithm for single-cut rotational osteotomy, a defined cutting angle was calculated for each specimen to achieve the proposed frontal alignment due to rotation. A self-designed 3D-printed cutting guide was used to provide the exact cutting angle.^29,32^ Preoperative and postoperative computed tomography (CT) scans and frontal-plane radiographs were obtained for the analysis of angles.

### Imaging Technique

**Figure 1.**
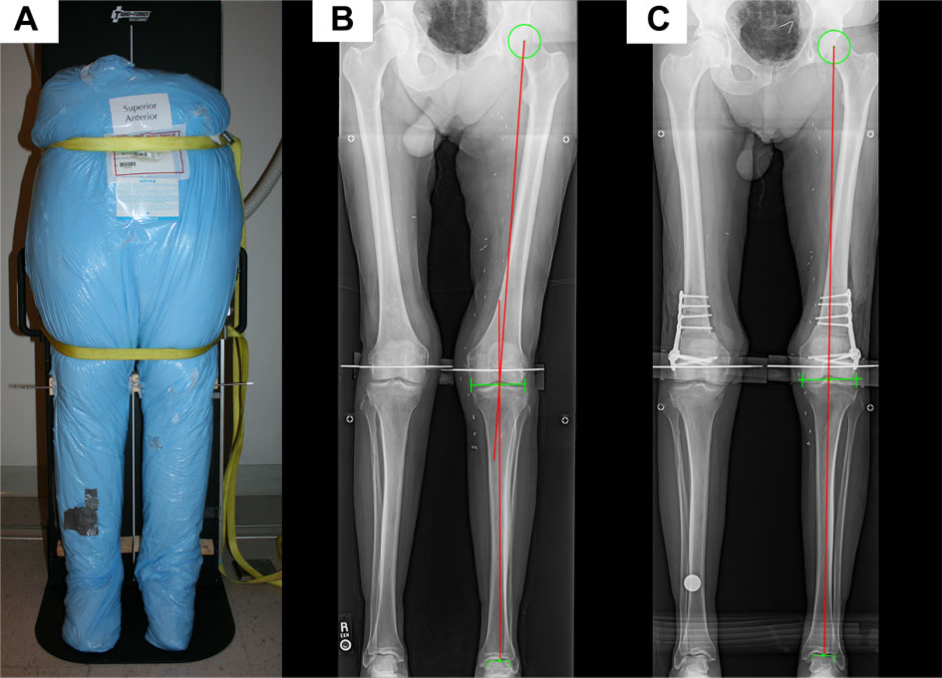
A thawed torso specimen: (A) strapped on an x-ray table in the upright position, (B) preoperative radiograph showing valgus alignment (red lines), and (C) postoperative radiograph showing straight alignment (red line) after derotation.

A standardized protocol for anteroposterior whole-leg radiographs and CT scans was developed. Upright whole-leg radiographs were obtained for alignment measurements preoperatively and postoperatively: After thawing, torso specimens were straightened and placed on an x-ray table in a supine position. After strapping the pelvis and middle of the femur, the x-ray table was erected to simulate a standing position (Figure 1). Frontal-alignment radiographs were considered adequate if the patellae were centered on the femoral condyles, as is done in clinics. For torsion measurements, CT scans were acquired with a standard bone window and using a kernel algorithm on a Somatom Definition dual-energy 64-slice scanner (Siemens), as done in clinics. With the torso specimen lying in a supine position, images from the iliac crest to the talus were reconstructed in the axial plane with a 2-mm slice thickness.^18,30^

### Imaging Measurements

Anatomic landmarks were chosen as described by Paley and Herzenberg.^22^ However, for reproducibility, landmarks were defined precisely as follows: The *mechanical femoral axis* was defined as a line from the center of the femoral head to the center of the distal femoral joint line. The *center of the distal femoral joint line* was defined as the midpoint on the tangent line of the most distal part of the femoral condyles and with regard to the most lateral and the most medial parts of each epicondyle. The *femoral anatomic axis* was drawn as a line between 2 points: (1) the midpoint between the greater trochanter and femoral neck, with a 3-point circle method on the proximal side, and (2) the midpoint of the shaft, 70 mm proximal to the knee joint line on the distal side. The *mechanical tibial axis* was drawn as a line from the middle of the tibial plateau to the middle of the proximal talus.

Valgus and varus alignment were determined as the mFTA according to Strecker.^26^ For the simplification and reproducibility of the measurement of this angle, the center of the femoral condyle joint line and the center of the tibial plateau joint line were averaged to serve as a single midpoint of the knee joint. A straight leg axis was determined as an mFTA of 0°, defining varus as positive values and valgus as negative values. The mechanical lateral distal femoral angle and anatomic mechanical angle (AMA) of the femur were collected as well. Preoperative measurements were performed by 1 surgery-independent author (A.V.) using mediCAD (version 4; Hectec). Preoperative planning was performed afterward by the surgeon (F.B.I.) on a DICOM viewer (OsiriX Lite v8.5.2; Pixmeo). Postoperative outcome measurements of angles were independently conducted by 3 observers (1 mediCAD, 2 OsiriX), and averaged values were taken.

**Figure 2.**
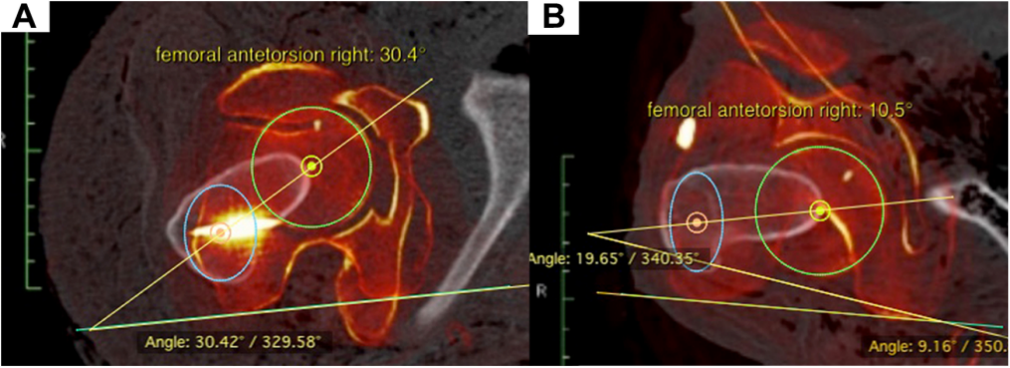
(A) Preoperative and (B) postoperative measurements of torsion with an axial slicing merging technique.

As described in several previous clinical studies, torsion measurements of the femur were performed using a CT-based method with axial slicing.^4,5,27^ The center of the femoral head was marked on the first image, and the center of the greater trochanter was marked on a second image with an elliptic circle. The third image showed a tangential line at the posterior condyles. When all 3 images were merged, the angle between the line from the femoral head to the greater trochanter versus the posterior condyles equaled femoral antetorsion (Figure 2) . Axial slicing fusion and measurements were performed with a DICOM viewer.

### Preoperative Planning

**Figure 3.**
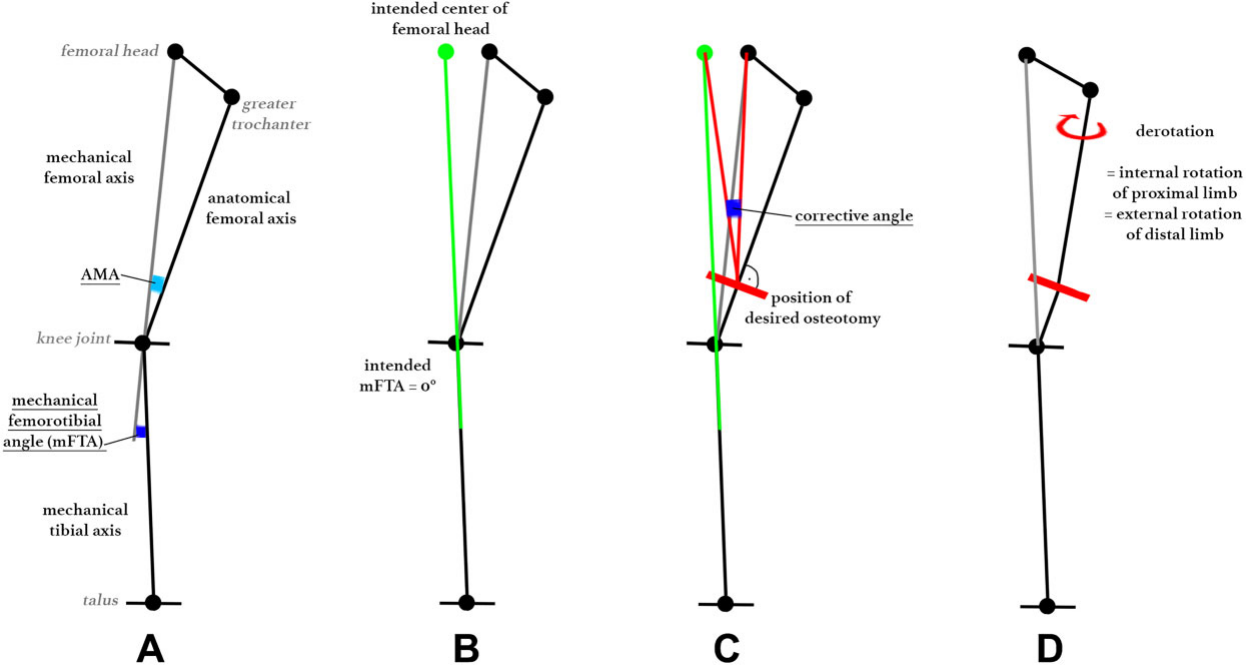
Stepwise preoperative planning for the corrective angle: (A) mechanical and anatomic femoral and tibial axes and angles, (B) intended center of the femoral head with an mFTA of 0°, (C) measuring the corrective angle at the intended cutting line, and (D) assumed change of the proximal femur because of derotation. AMA, anatomic mechanical angle.

**Figure 4.**
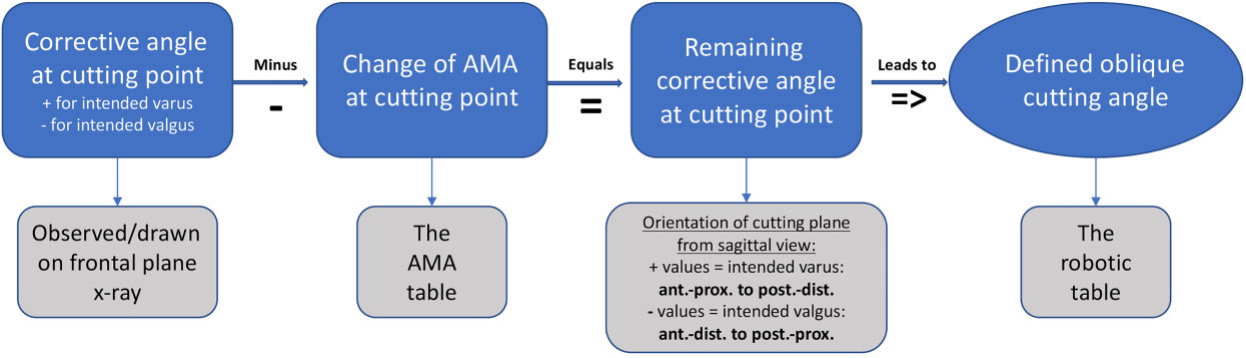
Process of the corrective angle to obtain the oblique cutting angle. AMA, anatomic mechanical angle.

The desired derotation of the distal femur was set to 20° in this cadaveric setup, as this represents an average value performed by the authors and in accordance with the current literature.^19,27,31^ Preoperative planning for additional frontal correction was performed stepwise as follows:

1. To achieve the desired mFTA of 0°, the corrective angle at the cutting point was drawn as described in Figure 3, according to Strecker.^26^ Intended varus (correction of valgus axis) were indicated by positive values, and intended valgus (correction of varus axis) were indicated by negative values.
2. Next, changes of the AMA at cutting because of derotation had to be taken into account: The observed AMA at the desired cut on a frontal radiograph measured at a certain femoral antetorsion angle led to an assumed slight increase (positive value) of the AMA because of derotation, described as the Pillar-Crane model.^14^ Changes of the AMA are provided in Appendix Table A1. The change of the AMA was subtracted from the corrective angle at the cutting point, which led to the remaining corrective angle at the cutting point (Figure 4).

### Mathematical Model

**Figure 5.**
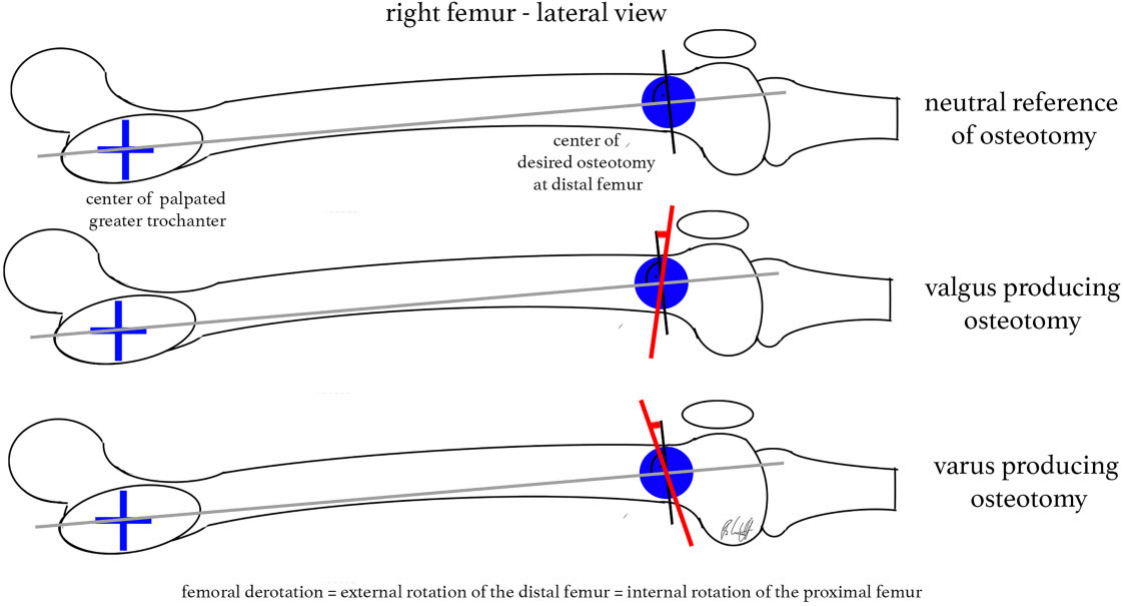
Correct inclination of the cutting plane: (A) verifying the virtual anatomic shaft axis from the lateral view, (B) inclination for a valgus-producing effect, and (C) inclination for a varus-producing effect.

The remaining corrective angle and the desired derotation angle equaled a defined oblique cutting angle. The cutting plane had to be inclined from the lateral view versus the virtual anatomic shaft axis (Figure 5).

To calculate the position of the rotated endpoint of the limb after a defined rotation angle and inclined cutting plane, an approach commonly used in robotics experiments was utilized. The Denavit-Hartenberg transformation matrix calculates, in a Cartesian coordinate system (*xyz* axes), the sagittal and coronal changes of the axis when defining an oblique cutting angle in one plane and rotation through its central axis.^11^ Trigonometric calculations were performed with Mathematica (version 11.1; Wolfram Research). An easy-to-read table was created and is shown in Appendix Table A2.

### Surgical Technique

**Figure 6.**
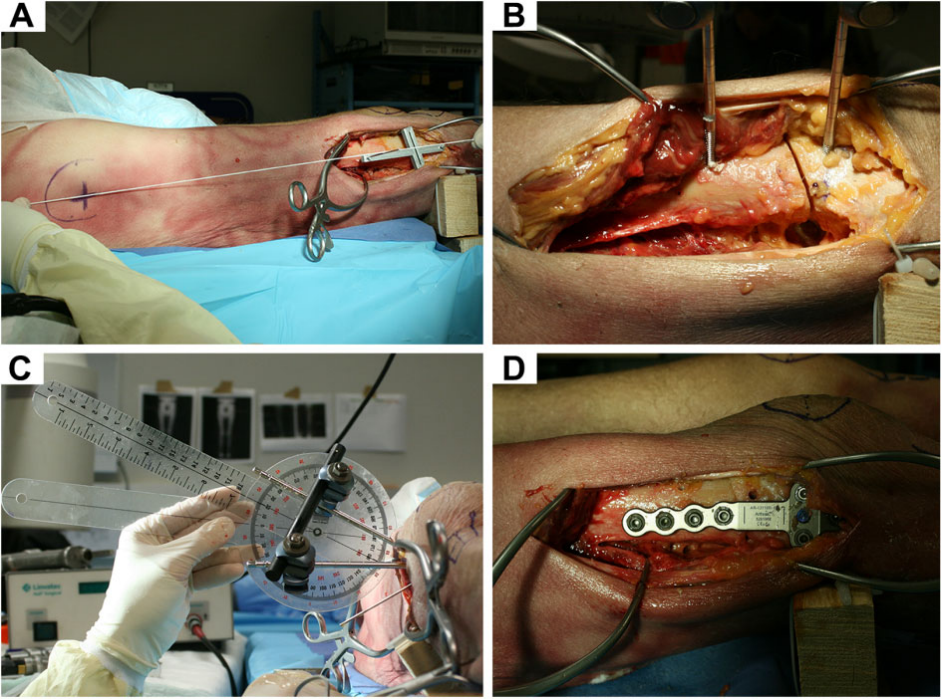
Procedure of distal femoral derotational osteotomy: (A) subvastus approach from the lateral view, identifying the virtual anatomic shaft axis with the 3-dimensional-printed cutting guide aligned; (B) single-cut osteotomy with an attached external fixator; (C) derotation observed with a goniometer; and (D) plate fixation of osteotomy.

For the surgical procedure, specimens were placed in a supine position on an operating room table. A lateral approach to the distal femur, as is commonly done with open-wedge techniques for distal femoral osteotomy, was performed.^13^ The 3D-printed cutting guide with a guiding slot was fixed above the femoral condyles with 2 K-wires. The 3D printing was performed before each surgical procedure on an Ultimaker2+ Extended machine (Ultimaker) out of polylactic acid material. The orientation of the device was meant to be parallel to the anatomic shaft axis from the frontal view and parallel to the virtual anatomic shaft axis with regard to the marked midpoint of the greater trochanter from the lateral view (Figure 6A). This simple guide did not reflect any specific anatomy of the bone. Then, an external fixator with 2 Steinmann pins was placed while the rail was not tightened. The position of the cut, that is, the midpoint of the oblique cut, was approximately 7 cm above the joint line according to the plate design and in accordance with preoperative planning, described as the position of the desired osteotomy. After osteotomy was performed (Figure 6B), derotation of 20° (external rotation of the distal femur = internal rotation of the proximal femur) was completed and observed with a goniometer from an axial perspective (Figure 6C), as is routinely done in clinical practice.^12^ The external fixator pins were used as joysticks for rotation, and the rail was then tightened to hold the osteotomy site in place while a distal femoral osteotomy locking plate (Arthrex) was applied (Figure 6D).

### Statistical Analysis

An a priori power analysis was performed based on the desired level of precision. A 95% CI with limits of ±1° selected as this level of precision was deemed clinically appropriate. An SD of 1° in alignment correction, as observed with goniometric measurements, was assumed across specimens. Using these estimates, a sample size of 8 specimens resulted in a 95% CI within ±1° of precision.

Descriptive statistics included the mean, SD, 95% CI, and range when applicable. Differences between preoperative and postoperative angles were examined with the paired *t* test and Wilcoxon signed-rank test (alpha level at 0.05). Angle values of varus or valgus axes, as well as corrective angles, were transcribed into positive angles for statistical comparison. Analyses were conducted with Stata/IC 14.2 (StataCorp).

## Results

Preoperatively, the mFTA ranged from –3.9° (valgus) to +3.4° (varus) (mean, –0.2° ± 2.6°). Postoperatively, the mean mFTA of 0.37° ± 0.69° (95% CI, –0.22° to 0.95°) was achieved. The change of axis, which was shown by the preoperative versus postoperative mFTA, was significant (paired *t* test: *P* = .01; Wilcoxon signed-rank test: *P* = .011). The mean intended correction of frontal alignment, determined as the corrective angle, was 2.6° ± 1.8° overall. The mean femoral antetorsion was 25.8° ± 6.0° (range, 19.0°-33.4°) preoperatively and 6.8° ± 5.4° (range, 0.0°-15.7°) postoperatively. Derotation showed a mean of 19.1° ± 2.1° (95% CI, 17.3°-20.8°). The AMA increased postoperatively by a mean of 0.30° ± 0.32° due to derotation. Detailed information is given in Table 1.

**TABLE 1:** Measurements of Cadaveric Specimens*^a^*

	Preoperative			Planning and Calculation			Cutting	Postoperative		
Specimen	mFTA*^b^*	AMA	Femoral Torsion	Corrective Angle	AMA Change Due to Derotation	Remaining Corrective Angle	Sagittal Cutting Angle*^c^*	mFTA*^b^*	AMA	Femoral Torsion
T1L	–3.9	5.2	19.0	4.5	0.3	4.2	12.4	0.5	5.6	3.5
T1R	–3.6	5.8	19.5	4.0	0.3	3.7	10.9	0.3	5.8	0.0
T2L	1.4	4.7	22.0	–2.2	0.3	–2.5	–7.3	0.8	5.2	4.3
T2R	3.4	4.9	22.3	–5.0	0.4	–5.4	–16.0	0.9	5.0	1.3
T3L	–0.6	5.5	26.5	0.6	0.6	0.0	0.0	1.1	5.3	7.6
T3R	–0.9	5.8	30.4	0.5	0.8	–0.3	–0.9	–0.7	6.3	10.5
T4L	0.7	4.1	33.3	–1.4	0.7	–2.1	–6.2	0.6	4.5	11.1
T4R	1.9	3.7	33.4	–2.9	0.6	–3.5	–10.3	–0.7	4.5	15.7

*^a^*All values are in degrees. AMA, anatomic mechanical angle; L, left; mFTA, mechanical femorotibial angle; R, right.

*^b^*Varus axis: +; valgus axis: –.

*^c^*Varus producing: +; valgus producing: –.

Subgroup analysis of the 4 valgus legs showed that the inclination of the cutting plane had to be between 0° and 12° (mean, 5.9° ± 6.8°) versus the perpendicular plane of the virtual anatomic shaft axis from the lateral view to achieve an additional frontal-alignment effect due to derotation. In this valgus subgroup, a mean change of 2.7° on the frontal axis was observed. Additional trigonometric calculations revealed a change on the sagittal axis by a mean increase of 0.3° of extension to the femur.

In the varus subgroup, the inclination of the cutting plane had to be –6° to –16° (mean, –10.0° ± 4.5°) to achieve frontal correction (mean change, –1.3°). Because of this amount of correction on frontal alignment, the sagittal change was calculated to be a –0.6° increase of extension, which equals an increase of flexion. According to the mathematical model, the length of the valgus and varus limbs shortened by only 0.2% (0.9-1.0 mm) of the femur length when derotation of 20° was performed at an inclined cutting angle of a mean of 5.9° and –10.0°, respectively.

## Discussion

The most important finding of this study is that single-cut derotational osteotomy of the distal femur can correct 2 angles (torsion and frontal alignment) according to preoperative calculations. The hypothesis was accepted within a clinically applicable SD of ±0.7° on postoperative frontal alignment and ±2.1° on derotation. This study serves as a proof of concept of a novel mathematical approach and its clinical feasibility, which explores a way to measure, plan, and perform derotational surgery of the distal femur.

Derotational osteotomy is a widely recognized and reliable procedure for the correction of femoral maltorsion. In 1996, Delgado et al^3^ described the technique of derotational osteotomy for the treatment of excessive femoral internal or tibial external torsion associated with patellofemoral abnormalities. Newer studies from Dickschas et al^5^ and Nelitz et al^19^ showed that distal femoral derotational osteotomy on even smaller antetorsion angles is an excellent treatment option in cases of patellofemoral maltracking as well. Unfortunately, while successful in treating antetorsion, rotational osteotomy carries a high risk of change of axis alignment.^16,20^ Paley and Herzenberg^22^ showed a way to address the frontal axis combined with torsion for the correction of deformities. Their mathematical approach is complex and contains information that can be adapted to different tubular bones. However, for a surgeon, it is very difficult to apply the correct cutting angle in vivo with regard to an anatomic landmark, especially at the femur, where mechanical and anatomic axes differ and bowing of the femur, known as antecurvation, occurs. Therefore, we investigated a new, simple, and reproducible approach, as the published techniques on derotational osteotomy of the distal femur have not included a guide for reference and orientation of the cut.^1,3,12,25,27^

We developed a novel mathematical approach and evaluated its practicability and reproducibility in a whole-leg cadaveric setup to address 3D changes on 2-dimensional imaging. Axial imaging was performed with a CT-based method by Waidelich et al,^30^ as is done in clinical studies. The frontal axis was measured and preoperative planning performed on upright whole-leg radiographs, as is done for the correction of valgus or varus alignment.

Orientations, plane views, and anatomic references due to rotation are elementary and not easily transferable into the operating room.^16^ With regard to derotational osteotomy in cases of patellofemoral instability, unintended axis malalignment such as postoperative increased valgus contradicts the initial purpose of treating patellofemoral malalignment. The presence of valgus malalignment is known to be an additional risk factor in patellofemoral instability cases, independent from maltorsion.^13,21,23,24^ Brattström^1^ stated that valgus alignment and muscular vectors on the anatomic femoral shaft may constitute additional potential factors in these cases. Furthermore, frontal malalignment contributes to the likelihood of patellofemoral osteoarthritis, as seen in large clinical observations.^2,7,17,28^

Our finding of a valgus-producing effect when the cutting plane is perpendicular to the distal shaft axis is consistent with the study from Nelitz et al.^20^ These authors stated that distal derotational osteotomy with perpendicular cuts led to increased valgus alignment compared with osteotomy of the proximal femur, as shown in their simulation model. We believe that this effect is primarily based on the orientation of the cut. Regarding the 4 valgus legs in our setup, our proposed model was able to prevent severe valgus malalignment due to derotational osteotomy of the distal femur. Therefore, we suggest referring to the virtual anatomic shaft axis in the operating room, which is a line from the center of the palpated greater trochanter to the center of the desired osteotomy site from the lateral view. In the operating room, with the patient placed in a supine position, the greater trochanter is marked in the lateral view to orient the surgeon to the desired cutting plane. Additionally, an oblique cutting angle toward the sagittal plane is reproducible and can be more easily verified when a lateral approach is performed than an angulation of the cut in the frontal plane. Furthermore, the mathematical model showed that an inclination of the cut from a lateral approach will have significantly more effect on the frontal axis than on the sagittal axis. If strong anterior bowing (sagittal axis) should additionally be corrected, the cutting plane will have to be oblique in the frontal plane as well.

### Limitations

Limitations of this work lie in the biomechanical setup of cadaveric specimens. We randomly received varus and valgus legs, and femoral torsion showed big differences. Therefore, some specimens underwent derotation toward a torsion angle of 0°, which is not clinically applicable. One could argue that femoral maltorsion and varus malalignment are rarely seen in clinics. However, for proof of concept of the mathematical approach, our model aimed to demonstrate its reproducibility and functionality regardless of preoperative torsion angles and frontal axes. A second limitation is that planning and surgery were performed by only 1 surgeon to obtain consistent data and prove the concept. Radiographic imaging was performed in an upright standing position and not a full weightbearing or supine position, which may lead to different measurements.

Another limitation is that increased femoral antetorsion and valgus malalignment in such cases often show dysplastic condyles and/or trochleas and a more curved femoral shaft. Our model did not address such pathological findings. However, from a mathematical point of view, our approach will theoretically achieve its proposed result when frontal radiographs are knee centered and axial CT slicing is performed with regard to the aforementioned landmarks. Therefore, further studies on dysplastic femoral anatomy will have to be conducted to show clinical applicability.

Furthermore, our study did not investigate greater amounts of the correction of frontal axis than 4°. The mathematical model does not have a limitation. However, with increasing frontal alignment correction (eg, 10°), the inclination of the defined cutting plane will increase as well (up to ≥30° oblique). We cannot comment on the biomechanical properties of plate fixation in such cases. Furthermore, derotational osteotomy does not correct the TT-TG distance, patella alta, or trochlear dysplasia, which can all contribute to patellar instability. Therefore, it is likely that derotational osteotomy will be performed in conjunction with other procedures.

The observed SD on torsion could be improved with the use of a real-time navigating tool, such as an electromagnetic tracking device as proposed by Geisbusch et al.^10^ The use of 3D-printed templates has been reported by Victor and Premanathan^29^ and Zheng et al^32^ to improve accuracy in osteotomy on the lower limb. The reduced use of fluoroscopy is an additional benefit. However, the presence of a 3D printer and correct plastic filament, the time-consuming creation of the templates, and the sterilizing process make this tool complex for normal clinical use.

## Conclusion

Distal femoral osteotomy can be performed to simultaneously address rotational as well as coronal alignment with a preoperatively defined single cut according to the trigonometric calculation model presented in this study.

## Appendix

**TABLE A1:** Changes of the AMA When Derotation of the Distal Femur Is Performed, Described as the Pillar-Crane Model^14^ *^a^*

Antetorsion	25°	30°	30°	35°	35°	40°	40°	40°	45°	45°	45°
Derotation	10°	10°	15°	15°	20°	20°	25°	30°	20°	25°	30°
AMA											
3.0°	0.2	0.3	0.3	0.4	0.5	0.7	0.8	0.9	0.8	1.0	1.1
3.5°			0.4	0.5	0.6	0.8	0.9	1.0	1.0	1.1	1.3
4.0°	0.3	0.3	0.5	0.6	0.7	0.9	1.0	1.1	1.1	1.3	1.5
4.5°			0.5	0.7	0.8	1.0	1.2	1.3	1.3	1.5	1.6
5.0°	0.3	0.4	0.6	0.7	0.9	1.1	1.3	1.4	1.4	1.6	1.8
5.5°			0.6	0.8	1.0	1.2	1.4	1.6	1.5	1.8	2.0
6.0°	0.4	0.5	0.7	0.9	1.1	1.3	1.5	1.7	1.7	2.0	2.2
6.5°			0.7	0.9	1.2	1.5	1.7	1.8	1.8	2.1	2.3
7.0°	0.5	0.6	0.8	1.0	1.2	1.6	1.8	2.0	1.9	2.3	2.5
7.5°			0.9	1.1	1.3	1.7	1.9	2.1	2.1	2.4	2.7

*^a^*All values are in degrees. Changes of the anatomic mechanical angle (AMA) indicate varus increase.

**TABLE A2:** Inclination of the Osteotomy When the Derotation Angle and Desired Change on the Frontal Axis Are Known*^a^*

Change on Coronal Axis	Derotation*^b^*						
	10°	15°	20°	25°	30°	35°	40°
1.0°	5.8	3.9	2.9	2.4	2.0	1.7	1.6
1.5°	8.7	5.8	4.4	3.6	3.0	2.6	2.3
2.0°	11.6 [0.2]	7.8	5.9	4.7 [0.4]	4.0	3.5	3.1 [0.7]
2.5°	14.5	9.7	7.3	6.0	5.0	4.4	3.9
3.0°	17.5	11.7	8.8	7.1	6.0	5.2	4.7
3.5°	20.6	13.6	10.3	8.3	7.0	6.1	5.4
4.0°	23.7 [0.3]	15.6	11.8	9.5 [0.9]	8.0	7.0	6.2 [1.4]
4.5°	26.9	17.6	13.3	10.7	9.0	7.9	7.0
5.0°	30.1	19.7	14.8	11.9	10.0	8.7	7.8
5.5°	33.5	21.7	16.3	13.1	11.0	9.6	8.6
6.0°	37.0 [0.4]	23.8	17.8	14.3 [1.3]	12.1	10.5	9.4 [2.2]
6.5°	40.7	26.0	19.3	15.5	13.1	11.4	10.1
7.0°	44.6	28.1	20.9	16.8	14.1	12.3	10.9
7.5°	48.7	30.3	22.4	18.0	15.1	13.1	11.7
8.0°	53.3 [0.4]	32.5	24.0	19.2 [1.7]	16.2	14.0	12.5 [2.7]
8.5°	58.4	34.8	25.6	20.5	17.2	14.9	13.3
9.0°	64.3	37.2	27.2	21.7	18.2	15.8	14.1
9.5°	71.9	39.6	28.8	23.0	19.3	16.7	14.9
10.0°	89.0 [0.01]	42.1	30.5	24.3 [2.0]	20.3	17.6	15.6 [3.5]

*^a^*All values are in degrees. Additional change on the sagittal axis is shown in brackets. Inclination of the cutting plane from the sagittal view: anterior-proximal to posterior-distal = varus producing; anterior-distal to posterior-proximal = valgus producing.

*^b^*Derotation = external rotation of the distal limb / internal rotation of the proximal limb.
